# The prognostic ability of radiotherapy of different colorectal cancer histological subtypes and tumor sites

**DOI:** 10.1038/s41598-023-38853-9

**Published:** 2023-07-20

**Authors:** Wenzai Shi, Jianfei Chen, Nan Yao, Tiantian Wu, Xiaopeng Suo, Qiang Wang, Jun Liu, Guoyong Yu, Keming Zhang

**Affiliations:** 1 Department of Hepatobiliary Surgery, Peking University International Hospital, Life Park Road No.1 Life Science Park of Zhong Guancun, Chang Ping District, Beijing, 102206 China; 2 Department of Surgery, Beijing Shijitan Hospital, Capital Medical University, Beijing, 100038 China; 3 Ninth School of Clinical Medicine, Peking University, Beijing, 100038 China; 4 School of Oncology, Capital Medical University, Beijing, 100038 China; 5 Department of General Surgery, Aerospace Center Hospital, Beijing, 100089 China; 6 Department of Nephrology, Beijing University of Chinese Medicine Affiliated Dongzhimen Hospital, East 4th North Street 279, Dongcheng District, Beijing, 100007 China

**Keywords:** Cancer, Psychology, Health care

## Abstract

The prognostic significance of radiotherapy (RT) for colorectal cancer (CRC) has shown conflicting results, particularly among different pathological subtypes, including adenocarcinoma (AC), mucinous adenocarcinoma (MC), and signet-ring cell carcinoma (SR). This study analyzed the prognosis of three pathological CRC types and focused on the prognostic significance of RT on three CRC histological subtypes. Patients diagnosed with AC (n = 54,174), MC (n = 3813), and SR (n = 664) in the National Cancer Institute’s Surveillance, Epidemiology, and End Results (SEER) database (2010–2017) were evaluated. Cox regression models and competitive risk models were built to assess the effect of RT on the risk of CRC-associated death. Potential interactions between RT and stratified variables including age, sex, and tumor location were examined by multiplicative models. Compared with AC patients, SR patients had the worst overall survival (OS) among 3 subtypes of CRC (log-rank test, p < 0.001). Compared with patients who did not receive radiotherapy, RT was associated with a 1.09-fold (HR = 1.09, 95%[CI]: 1.03, 1.15) elevated risk of death among AC patients. In the SR group, RT significantly reduced the risk of death by 39% (HR = 0.61, 95%[CI]: 0.39–0.95). However, RT did not appear to independently influence survival in the MC group (HR = 0.96, 95%[CI]: 0.77, 1.21). In the subgroup analysis, tumor location (colon and rectum) significantly modified the association between RT and the risk of death among the AC and SR patients (p for interaction < 0.05). SR patients exhibited a worse OS (overall survival) than AC patients, and the effect of RT varied according to CRC histological subtypes. This can ultimately lead to more personalized and effective treatment strategies for CRC patients.

## Introduction

Colorectal cancer (CRC) is one of the most frequent cancers in the world. According to the 2021 American cancer statistics^1^, approximately 149,500 new CRC cases and 52,980 deaths were expected to occur, accounting for approximately 10% of cancer cases and deaths. Notably, the 2021 projections are based on currently available incidence and mortality data and thus do not reflect the impact of COVID-19 on cancer cases and deaths. Overall, CRC (colon and rectum cancers) ranks fourth in terms of incidence, with 104,270 colon cancer (both sexes) and 45,230 rectum cancer cases (both sexes), and it is the second leading cause of cancer-related deaths (a large number of deaths of rectal cancer are misclassified as colon cancer, nonetheless both cancers have a large number of deaths: a combined estimation of 52,920). Despite advances in CRC detection, diagnosis, and treatment, it remains a serious threat to people’s lives and health.

CRC is characterized by several histological subtypes, with adenocarcinoma (AC) being the most frequent type^2–4^. Mucinous adenocarcinoma (MC) and signet-ring cell carcinoma (SR) are thought to represent separate subgroups that account for 4–30% and 1% of all colorectal malignancies, respectively. The survival in these three types of CRC is inconsistent^5–8^. Results from several studies showed MC and SR had a worse prognosis compared with AC^5,7–9^, however, other studies have found no difference in survival outcomes when compared to AC^10,11^.

Treatment strategies for CRC include surgery, chemotherapy, radiotherapy (RT), targeted therapy and immunotherapy. Surgery, chemotherapy, and radiotherapy are the three most commonly used method in clinical practice, and the implications as well as benefits of the first two have been well-studied previously^12–16^. However, the prognostic significance of RT for CRC has been conflicting, especially among different pathological subtypes. A prior study found that RT did not affect overall survival in either AC or MC patients^17^. In addition, a previous study has found cell subpopulations exhibiting intrinsic radio-resistance persist following RT, potentially leading to increased clinical risks due to their revived proliferation and successful colonization at local or distant sites^18^. Xu et al.^19^, on the other hand, discovered that CRC patients with MC/SR could benefit more from surgery combined with radiotherapy than surgery alone. There has been no large-scale population-based investigation focusing on the survival benefit of RT in distinct histological subtypes of CRC.

In the current study, we used data from the National Cancer Institute’s Surveillance, Epidemiology, and End Results (SEER) database from 2010 to 2017 to assess the prognosis and survival benefit of RT in CRC patients with AC, MC, and SR.

## Materials and methods

### Study participants

**Figure 1:**
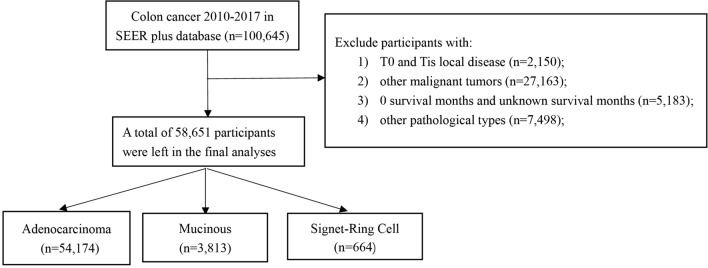
Flow chart of study participants.

We analyzed CRC patients registered in the SEER national cancer registry from 2010 to 2017. The SEER database, an authoritative source of information concerning cancer incidence and survival in America, is a population-based cancer registry. It records information on patients’ clinicopathological characteristics, the information on adjuvant therapy, and their vital status during follow-up. From 2010 SEER began to collect data on chemotherapy, radiotherapy and other important variables. In short, a total of 100,645 CRC patients were identified in the SEER database from 2010 to 2017. Patients were excluded if they (1) had T0 or Tis local disease (n = 2150); (2) were combined with other primary malignant tumors to exclude the impact of other cancer types (n = 27,163); (3) had a survival time of 0 months or uncertain survival months (n = 5183); and (4) had other pathological categories, except AC, MC and SR (the most common pathological types) (n = 7498). Finally, 58,651 patients were included in the final analysis (54,174 AC patients, 3813 MC patients, and 664 SR patients) (Fig. 1).

### Variables

Trained registrars systematically gather data on patients of all ages across clinical facilities involved in cancer diagnosis or treatment, without any discrimination based on insurance status. The information collected includes the date and cause of death, which is cross-referenced with death certificates. The U.S. Census Bureau data is utilized to calculate comprehensive death statistics. The demographic and tumor characteristics included sex, age, race, T stages, axillary lymph node status, liver, bone, brain, and lung metastases, perineural invasion, histological grades, and carcinoembryonic antigen (CEA), surgery, chemotherapy (CT), and radiotherapy (RT). CT and RT (including external-beam radiation, radioactive, implants, radioisotopes, or other types of radiation) were defined as received CT (RT) at any point of treatment. Patients who did not receive CT (RT) or with missing data on CT (RT) were categorized as not received CT (RT) or unknown, since the SEER database included “No” and “Unknown” in the chemotherapy and radiotherapy grouping. In accordance with previous studies, variables that had missing data were classified as “Unknown” for analysis purposes^20,21^.

### Statistical analysis

All statistical analyses were performed using a commercially accessible software program (SAS software, version 9.4). Continuous variables were described and compared using means ± standard deviations and one-way analysis of variance (ANOVA) or t-test based on the grouping method. Categorical variables were expressed as absolute values with percentages and were compared using the chi-square test. To examine the differences in survival probability over time across the three groups (overall and specific sites including colon and rectum), Kaplan–Meier survival curves were employed, and the equality of these curves was assessed using a log-rank test. The Nelson-Aalen cumulative risk curve was used to compare distributions of time to CRC-related death because CRC unrelated death was a competing risk event.

Person-years were calculated starting from the date of cancer diagnosis, to the date of CRC-related death, non-CRC-related death, or the date utilized as a cutoff for our analysis (December 31, 2017), whichever occurred first. The Schoenfeld residual test was used to examine the proportional hazard assumption. Cox regression models were used to describe the association between RT and the risk of CRC-associated death among different CRC pathological types. Three models were fitted as follows: model 1 was an unadjusted analysis; model 2 was adjusted for sex and age (every 10 years); model 3 was further adjusted for race, location, grades, T, N, and M stages, surgery, CEA, perineural invasion, and CT based on model 2. Subgroup analyses were carried out for three pathological categories of CRC that were stratified by age, tumor sites, and sex. The interactions between RT and these variables were further tested using multiplicative models.

During the follow-up, other causes of failure may arise before the cancer event, making it difficult to detect the presence of incident cancer. Because people who have a competing event (and hence are censored) are thought to develop cancer in the future, traditional predictions such as Cox regressions may significantly overestimate the cancer risk in this competing risk setting^22^. In that case, the competing risk analysis, the cause-specific hazard (CS) model and the sub-distribution proportional hazards (SD) models, were utilized to quantify the risk of CRC-related death, it allows for the estimation of event-specific cumulative incidence functions, providing a more accurate representation of the probabilities of each event occurring in the presence of competing risks. We further employed propensity score matching (PSM) analysis to mitigate selection bias and enhance the evidential strength of our nonrandomized observational study. Statistical significance was defined as a two-sided p value less than 0.05.

### Ethics approval and consent to participate

The present study used previously collected anonymized and de-identified data from the SEER database. Therefore, no additional informed consent was required. The study was complied with the 1964 Helsinki Declaration and its later amendments or comparable ethical standards and deemed exempt from review by the Ethics Board of the Peking University International Hospital.

## Results

The demographics and tumor characteristics of AC, MC, and SR patients are shown in Table 1. The mean age of MC patients (64.63 ± 14.47 years) was similar to that of AC patients (64.37 ± 13.46 years), however, SR patients were significantly younger (62.10 ± 15.77, *p* < 0.001). MC (63.73%) and SR (61.75%) were more commonly found in the right colon than AC (38.45%, *p* < 0.001). Moreover, SR was more likely to present with later stage lesions (stage III/IV, 60.85%) than MC (44.27%, p < 0.001) and AC (36.43%, p < 0.001). In addition, SR patients were more likely to receive chemotherapy than MC patients and AC patients. The percentage of race, marital status, sex, T, N, and M stages, CEA, perineural invasion, liver, bone, brain, and lung metastases, surgery, and radiotherapy differed significantly across the three groups. We also analyzed the baseline characteristics of the population stratified with and without radiotherapy for each pathological type (Supplementary Table 1).

**Table 1:** Baseline characteristics of the population stratified by pathological types.

	Adenocarcinoma n = 54,174	Mucinous n = 3,813	Signet-ring cell n = 664	*p*-value
Age (year)	64.37 ± 13.46	64.63 ± 14.47	62.10 ± 15.77	< 0.001
Race (%)				< 0.001
White	40,439 (74.65)	3016 (79.10)	525 (79.07)	
Black	6434 (11.88)	397 (10.41)	67 (10.09)	
Others	7301 (13.48)	400 (10.49)	72 (10.84)	
Marital status (%)				0.007
Single	9092 (16.78)	633 (16.60)	147 (22.14)	
Married	28,442 (52.50)	2020 (52.98)	332 (50.00)	
Other	16,640 (30.72)	1160 (30.42)	185 (27.86)	
Sex (%)				< 0.001
Female	25,521 (47.11)	1955 (51.27)	326 (49.10)	
Male	28,653 (52.89)	1858 (48.73)	338 (50.90)	
Location (%)				< 0.001
Right	20,831 (38.45)	2430(63.73)	410 (61.75)	
Left	15,047 (27.78)	662 (17.36)	106(15.96)	
Rectum	16,961 (31.31)	592 (15.53)	123 (18.52)	
Unknown	1335 (2.46)	129 (3.38)	25 (3.77)	
Grade (%)				< 0.001
Well-differentiated	3891 (7.18)	457 (11.99)	6 (0.90)	
Moderately-differentiated	35,816 (66.11)	2122 (55.65)	31 (4.67)	
Poorly-differentiated	6802 (12.56)	576(15.11)	414 (62.35)	
Undifferentiated	1193 (2.20)	179 (4.69)	99 (14.91)	
Unknown	6472 (11.95)	479 (12.56)	114 (17.17)	
AJCC 7th, T stage (%)				< 0.001
T1	7690 (14.20)	130 (3.41)	27 (4.07)	
T2	4749 (8.77)	254 (6.66)	22 (3.31)	
T3	18,248 (33.68)	1402 (36.77)	158 (23.80)	
T4	5957 (11.00)	949 (24.89)	247 (37.20)	
Unknown	17,530 (32.36)	1078 (28.27)	210 (31.63)	
AJCC 7th, N stage (%)				< 0.001
N0	21,906(40.44)	1507 (39.52)	152 (22.89)	
N1	10,925 (20.17)	743 (19.49)	113 (17.02)	
N2	5240 (9.67)	559 (14.66)	210 (31.63)	
Unknown	16,103 (29.72)	1004 (26.33)	189 (28.46)	
AJCC 7th, M stage (%)				< 0.001
M0	31,505 (58.16)	2130 (55.86)	299 (45.03)	
M1	8430 (15.56)	793 (20.80)	211 (31.78)	
Unknown	14,239 (26.28)	890 (23.34)	154 (23.19)	
Stages				< 0.001
I	9528 (17.59)	287 (7.53)	24 (3.61)	
II	9437 (17.42)	910(23.87)	68 (10.24)	
III	11,308 (20.87)	895 (23.47)	193 (29.07)	
IV	8430 (15.56)	783 (20.80)	211 (31.78)	
Unknown	15,471 (28.56)	928 (24.34)	168 (25.30)	
CEA (%)				< 0.001
Negative	17,466 (32.24)	1053 (27.62)	177 (26.66)	
Positive	15,733 (29.04)	1269 (33.28)	251 (37.80)	
Unknown	20,975 (38.72)	1491 (39.10)	236 (35.54)	
Perineural invasion (%)				< 0.001
Negative	38,640 (71.33)	2523 (66.17)	287 (43.22)	
Positive	4898 (9.04)	293 (7.68)	124 (18.67)	
Unknown	10,636 (19.63)	997 (26.15)	253 (38.10)	
Liver metastases (%)				< 0.001
No	44,557 (82.25)	3342 (87.65)	600(90.36)	
Yes	8541 (15.77)	407 (10.67)	49 (7.38)	
Unknown	1076 (1.99)	64 (1.68)	15 (2.26)	
Bone metastases (%)				0.003
No	52,340 (96.61)	3710 (97.30)	637 (95.93)	
Yes	616 (1.14)	36 (0.94)	16 (2.41)	
Unknown	1218 (2.25)	67 (1.76)	11 (1.66)	
Brain metastases (%)				0.020
No	52,772 (97.41)	3742 (98.14)	652 (98.19)	
Yes	172 (0.32)	5 (0.13)	3 (0.45)	
Unknown	1230 (2.27)	66 (1.73)	9 (1.36)	
Lung metastases (%)				< 0.001
No	49,927 (92.16)	3605 (94.54)	635 (95.63)	
Yes	2959 (5.46)	134 (3.51)	16 (2.41)	
Unknown	1288 (2.38)	74 (1.94)	13 (1.96)	
Surgery (%)				< 0.001
No	8783 (16.21)	370 (9.70)	148 (22.29)	
Yes	45,248 (83.52)	3437 (90.14)	515 (77.56)	
Unknown	143 (0.26)	6 (0.16)	1 (0.15)	
Chemotherapy				< 0.001
No/Unknown	29,095 (53.71)	1869 (49.02)	240 (36.14)	
Yes	25,079 (46.29)	1944 (50.98)	424 (63.86)	
Radiotherapy				< 0.001
No/Unknown	45,089 (83.23)	3408 (89.38)	574 (86.45)	
Yes	9085 (16.77)	405 (10.62)	90 (13.55)	

Note: The definitions of T, N, M were referred to as pathologic stage groups (pTNM). CEA, carcinoembryonic antigen.

**Figure 2:**
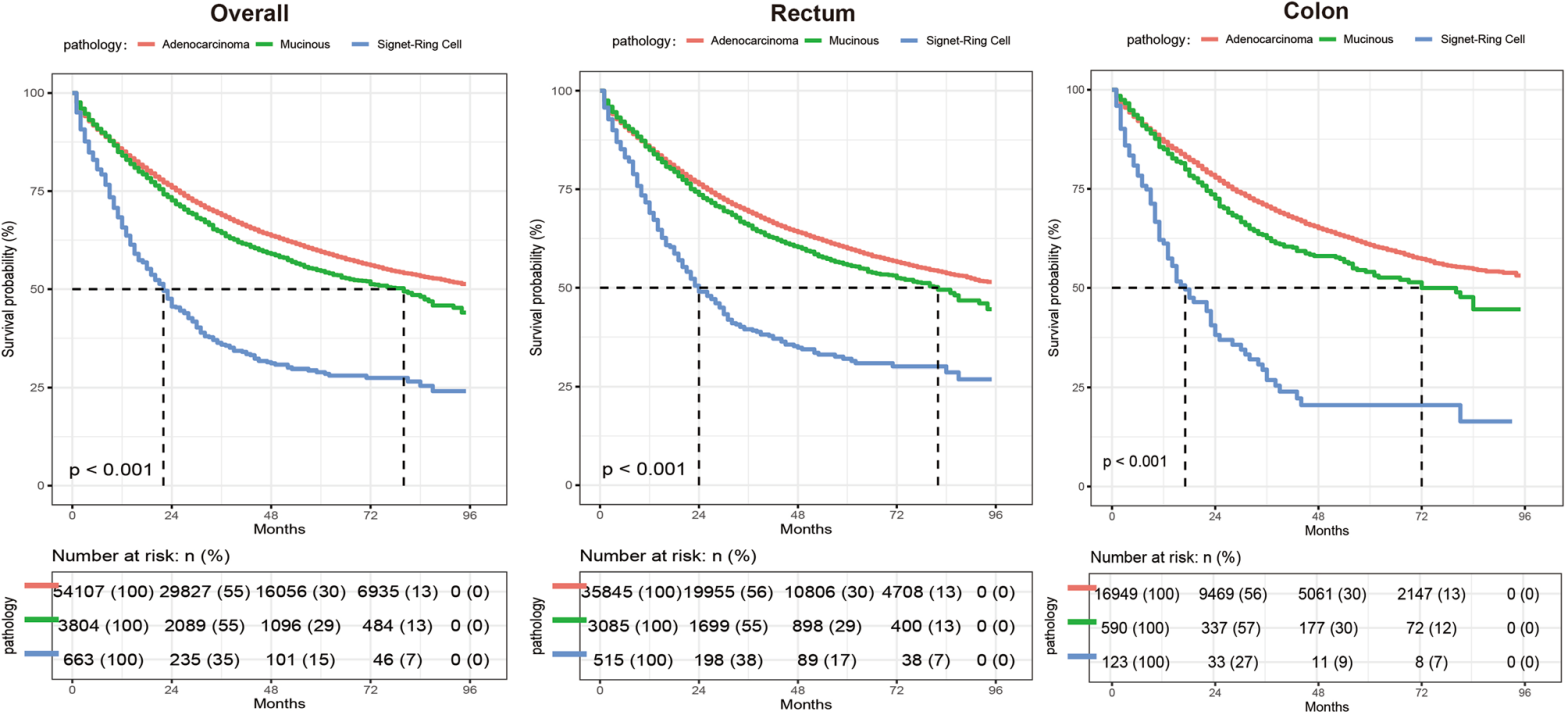
Overall survival among the AC, MC and SR groups.

**Figure 3:**
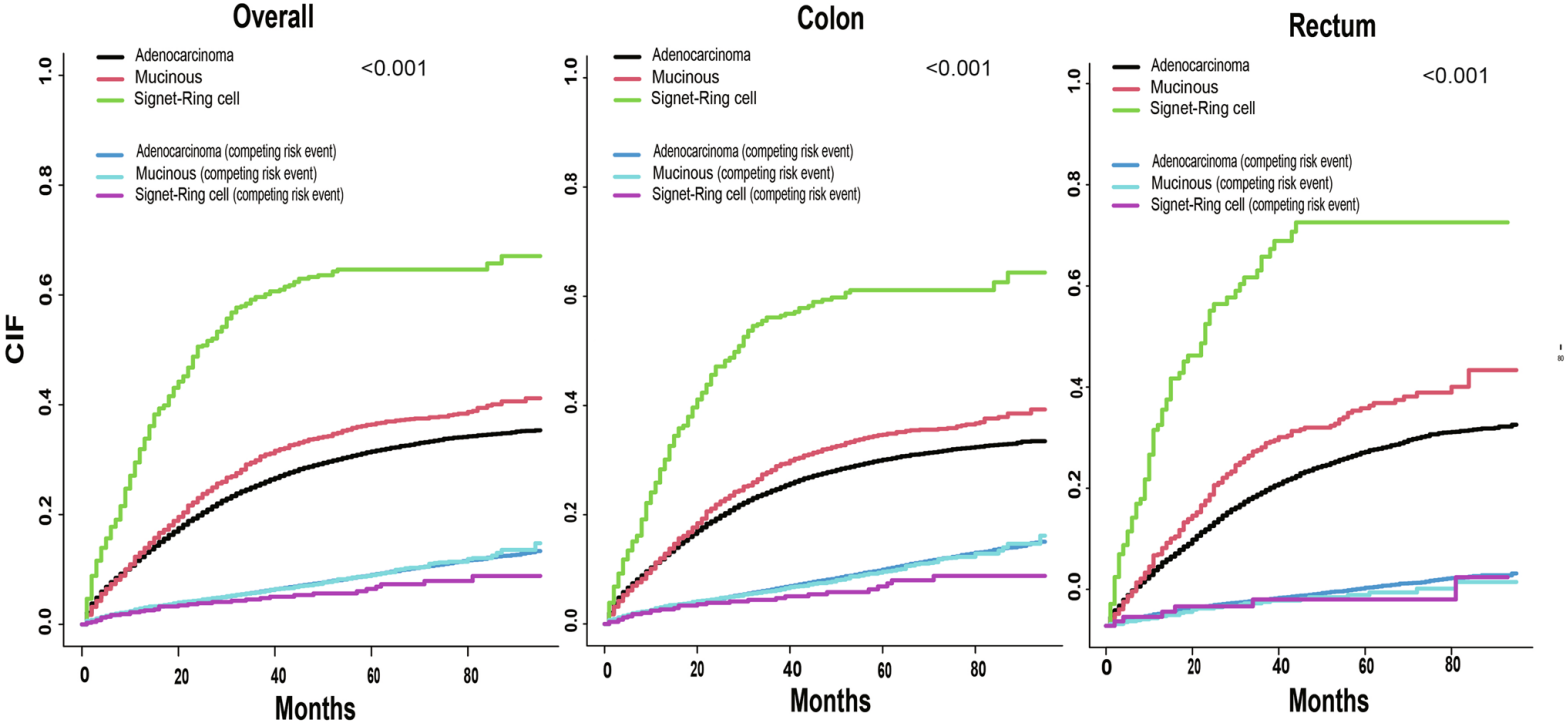
Cumulative incidence functions (CIFs) of CRC related death and the competing risk event based on the adenocarcinomas, MC and SR groups.

During the median follow-up of 27 (11–53) months among 58,651 participants, 14,973 CRC patients died (13,500 in AC group, 1113 in MC group, 360 in SR group). In addition, 4166 non-CRC-related death cases were observed before the occurrence of CRC-related death (3843 in AC group, 287 in MC group, 36 in SR group). Results from the Kaplan–Meier method (Fig. 2) showed that SR patients (overall, colon and rectum cancer) had the worst overall survival (OS) among 3 subtypes of CRC (log-rank test, p < 0.001). Significant differences were observed in the CRC related death rate among three histological subtypes (Fig. 3). Results from the Schoenfeld residual global test showed there was no violation of the proportional hazard assumption (p = 0.322). Table 2 demonstrates the association of the different pathological types with the risk of overall death. Compared with AC patients, SR patients were associated with a 1.28-fold (HR = 1.28, 95% CI: 1.16–1.42) risk of death, but failed to find such an association of MC with risk of death when compared with AC patients (HR = 0.99, 95% CI: 0.94–1.05).

**Table 2:** The association of pathological types with CRC-specific survival.

Pathological types	Univariate analyses		Multivariate analyses	
	HR (95%CI)	*p*-value	HR (95%CI)	*p*-value
Adenocarcinoma	Ref.		Ref.	
MC	1.15 (1.09, 1.21)	< 0.001	0.99 (0.94, 1.05)	0.819
SR	2.48 (2.25, 2.74)	< 0.001	1.28 (1.16, 1.42)	< 0.001

Note: Multivariate analyses were adjusted for age, sex, race, marital status, tumor location, grades, TNM stages, surgery, CEA, perineural Invasion, chemotherapy, and radiotherapy.

Table 3 describes the association between RT and the risk of death stratified by different pathological types. The Schoenfeld residual global test found no violation of the proportional hazard assumption in the AC (p = 0.122), MC (p = 0.317) and SR group (p = 0.091). Compared with patients without radiotherapy, RT was associated with a 1.09-fold (HR = 1.09, 95%[CI]: 1.03,1.15) elevated risk of death among AC patients. In the SR group, RT significantly reduced the risk of death by 39% (HR = 0.61, 95%[CI]: 0.39–0.95). However, RT did not appear to independently influence survival in the MC group (HR = 0.96, 95%[CI]: 0.77, 1.21). During the follow-up, a total of 4,166 non-CRC-related deaths were observed before the occurrence of CRC-related deaths. After considering non-CRC-related death, the CS models did not substantially alter the conclusions and even strengthened the HR from 1.09 (1.03–1.15) to a higher level (HR = 1.11, 95%[CI]: 1.05–1.17) among participants with adenocarcinoma CRC and from 0.61(0.39,0.95) to 0.60 (0.38–0.95) among the SR group. Similar results were also found in the SD models with minor differences (Table 3).

**Table 3:** The association of radiotherapy with the risk of death among CRC patients stratified by pathological types in different regressions.

	Adenocarcinoma		Mucinous		Signet-ring cell	
	HR (95%CI)	*p*-value	HR (95%CI)	*p*-value	HR (95%CI)	*p*-value
Cox regressions						
Model 1	0.86 (0.82, 0.89)	< 0.001	0.84 (0.70, 1.00)	0.050	0.92 (0.69, 1.23)	0.580
Model 2	1.00 (0.96, 1.05)	0.915	0.94 (0.79, 1.12)	0.480	0.96 (0.71, 1.29)	0.784
Model 3	1.09 (1.03, 1.15)	0.001	0.96 (0.77,1.21)	0.751	0.61 (0.39, 0.95)	0.028
CS models						
Model 1	0.94 (0.90, 0.99)	0.009	0.91 (0.75, 1.10)	0.332	0.94 (0.70, 1.26)	0.667
Model 2	1.03 (0.99, 1.08)	0.175	0.95 (0.78, 1.15)	0.599	0.94 (0.69, 1.28)	0.702
Model 3	1.11 (1.05, 1.17)	0.001	0.91 (0.71, 1.16)	0.429	0.60 (0.38, 0.95)	0.029
SD models						
Model 1	0.96 (0.92, 1.00)	0.067	0.93 (0.77, 1.11)	0.420	0.95 (0.72, 1.24)	0.681
Model 2	1.04 (0.99, 1.09)	0.108	0.95 (0.79, 1.15)	0.608	0.93 (0.71, 1.22)	0.611
Model 3	1.08 (1.02, 1.15)	0.011	0.87 (0.67, 1.12)	0.280	0.59 (0.36, 0.94)	0.020

Note:

Model 1: Univariate analysis.

Model 2: Adjusted for age and sex based on model 1.

Model 3: Further adjusted for race, location, grades, T, N, and M stages, surgery, CEA, perineural invasion, and chemotherapy based on model 2.

CS model: cause-specific hazard model.

SD model: sub-distribution proportional hazards model.

Table 4 shows the association of radiotherapy with the risk of death among CRC patients stratified by pathological types by using the PSM analysis. Participants who did not receive RT were chosen as controls, and they were matched by age (± 1 year), sex, tumor grades, stages, in a 1:1 ratio. A total of 19,160 participants were left in the PSM analysis (Supplementary Fig. 1). Similar results with the main findings were also obtained, which RT elevated the risk of death among AC patients (HR = 1.25, 95%[CI]: 1.17–1.35), and decreased the risk of SR patients (HR = 0.63, 95%[CI]: 0.41–0.97).

**Table 4:** The association of radiotherapy with the risk of death among CRC patients stratified by pathological types by using propensity score matching analysis (9580 participants received RT vs. 9580 participants did not receive RT).

	Adenocarcinoma		Mucinous		Signet-ring cell	
	HR (95%CI)	*p*-value	HR (95%CI)	*p*-value	HR (95%CI)	*p*-value
Model 1	0.75 (0.72, 0.79)	< 0.001	0.78 (0.64, 0.95)	0.013	0.89 (0.64, 1.22)	0.465
Model 2	0.78 (0.74, 0.82)	< 0.001	0.79 (0.65, 0.96)	0.019	0.91 (0.64, 1.28)	0.742
Model 3	1.25 (1.17, 1.35)	< 0.001	1.07 (0.80, 1.43)	0.646	0.63 (0.41, 0.97)	0.029

Note:

Model 1: Univariate analysis.

Model 2: Adjusted for age and sex based on model 1.

Model 3: Further adjusted for race, location, grades, T, N, and M stages, surgery, CEA, perineural invasion, and chemotherapy based on model 2.

**Figure 4:**
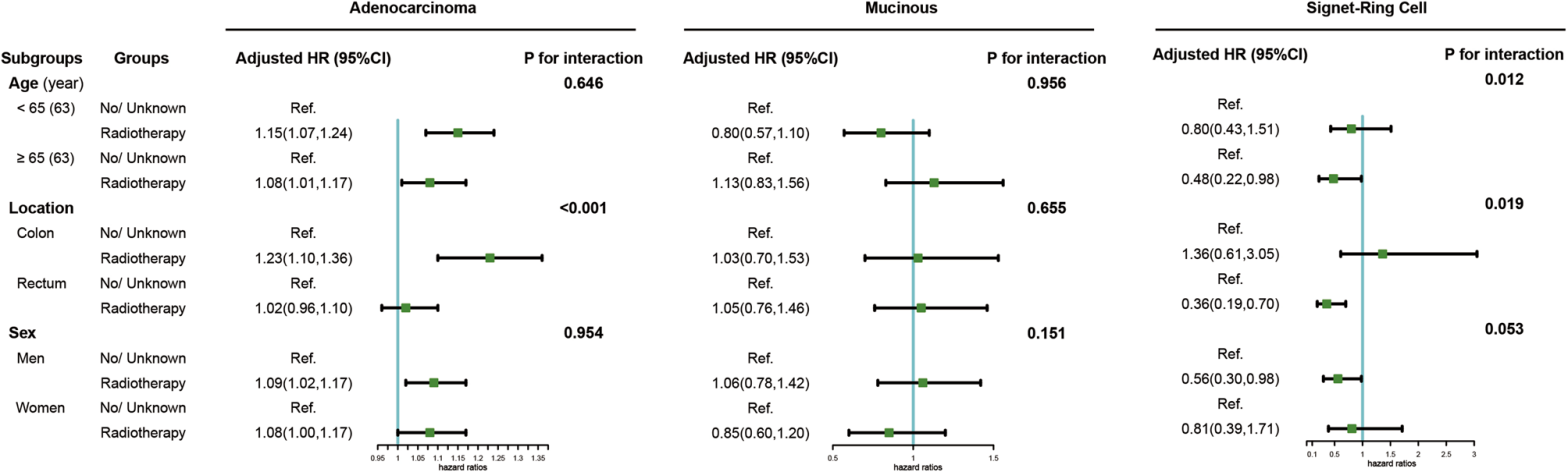
Subgroup analyses of the association of RT with the risk of death stratified by CRC histological subtypes.

Figure 4 shows the subgroup analyses by age, tumor location, and sex. The positive associations of RT with the risk of death were observed among AC patients who were elderly, male, and with the tumor located in the colon. Notably, tumor location affected the association between RT and the risk of death in the AC group. Moreover, the reversed associations of RT with death risk were found among SR patients who were older, male, and with a tumor located in the rectum. Similarly, the age and tumor location significantly revised the association of the SR group.

## Discussion

In this large population-based cohort study, we discovered that (1) SR patients had the worst OS of the three subtypes of CRC; (2) RT significantly increased the risk of death among patients with adenocarcinoma CRC, particularly among patients who were elderly, male, and had a tumor in the colon; and (3) RT significantly reduced the risk of death in SR patients, particularly among patients who were older, male, and with the tumor located the rectum.

The current study’s findings of poor prognosis in SR patients were in line with the majority of previous research. In a Korean study^5^, the 5-year survival rate in SR patients was only 26.8%, which was significantly worse than MC (58.1%, p < 0.001) and AC (62.9%, p < 0.001). By analyzing 244,794 CRC patients from the National Cancer Data Base (NCDB), Hyngstrom et al.^11^ found patients with signet ring cells colorectal AC had poorer outcomes compared with non–signet ring AC of either the colon or rectum. The association of MC with the risk of mortality of CRC patients was controversial. According to a population-based study in Singapore^7^, MC is an independent prognosis factor for poorer outcomes of CRC-related death. Whereas, other studies found that poor MC outcomes were more related to tumor stage or specific tumor sites, suggesting that MC is not an independent prognostic factor^23,24^.

RT was discovered to be a risk factor for AC patients’ prognosis in this study. Subgroup analyses further confirmed the elevated risks of RT for the prognosis of AC patients were observed among patients with colon cancer, however, not with rectum cancer. Several previous studies partly supported our results. A randomized controlled trial (RCT) involving 1861 CRC patients conducted by Kapiteijn et al.^25^ found the overall survival rate among eligible patients at two years was 82.0% in the group receiving both radiation and surgery and 81.8% in the group receiving surgery alone (p for difference = 0.840). Also, results from the randomized multicenter TME trial observed there was no improvement in survival of 10-year OS for AC patients (53.3% for surgery alone vs. 50.5% for surgery after radiotherapy; p for differences = 0.679)^17^. Unfortunately, no existing studies found that RT elevated the risk of death among AC patients with colon cancer. However, the advantages of RT should be noted as well. RT could reduce the incidence of local recurrence, down-stage locally advanced cancer, and increased resection rate for tumors, as well as raise the likelihood of anus reservation^26–28^. Despite the therapeutic effects of radiation being primarily attributed to DNA double-stranded breaks, a subset of cancer cells with intrinsic radio-resistance can survive this treatment. Reactivation of proliferation in these cells and their successful colonization at local or distant organs can lead to local regrowth and distant metastasis, increasing the risk of poor clinical outcomes. Recent studies have observed a more aggressive phenotype in radioresistant cancer cells, with altered gene expression related to cell cycle progression, DNA damage repair, migration, and invasion^29–31^.

We did not find a significant association between RT and OS in MC patients which was similarly shown in a prior study. Importantly, RT significantly lowered the risk of death for SR patients, particularly when the tumor was situated in the rectum rather than the colon. Consistent with our findings, Ling and his colleagues found that RT was an independent prognostic factor associated with improved survival in stage III rectal SR, rectal patients with stage III underwent preoperative RT had a better prognosis than patients who received surgery alone (HR = 0.47, 95% CI: 0.27–0.79)^32^. Furthermore, Guan et al.^19^ discovered that RT might improve survival in patients in stages II and III by evaluating data from 1808 rectum cancer patients in the SEER database, which is corroborated by another research by Wu^33^.

We provided evidence that RT is closely associated with worse prognosis among AC patients and better prognosis in SR patients was not yielded to analytical methods that consider the competing risk of cancer-free death. The occurrence of the event of interest is frequently prevented by another event in time-to-event studies, making it difficult to observe the occurrence of the event of interest. During an average follow-up of 27 months, 4166 people died from causes other than CRC. Compared to the results of Cox regression, the results were slightly strengthened in the competitive risk approaches, confirming the primary conclusions of the current study. In the current study we applied both the CS models and the SD models. Despite comparable conclusions, the CS model and SD model are distinct. The CS model would be more appropriate for investigating the causal association. The SD model would be more suited to predicting the likelihood of the result^34^.

The underlying mechanism remains uncertain. However, several mechanisms may help to explain our findings. First, the expression of the p53 protein was found to be higher in the AC component of the tumor than in the signet ring carcinoma component^35^. The presence of wild-type p53 in cancer cells has been linked to susceptibility to radiation or chemotherapy-induced damage, whereas mutant p53 has been linked to radio- and chemotherapeutic resistance^36^. Second, SR cancer showed significantly negative/lower expression of epidermal growth factor receptor (EGFR), compared with other pathologic types of CRC^37^. EGFR regulates a variety of physiological responses, including cell proliferation, apoptosis, and differentiation, and is suspected to have a role in a variety of cancers. In in vitro tests of malignant cell research, overexpression of EGFR has been linked to radio-resistance^38,39^. Third, the expression of cyclooxygenase (COX)-2 was reduced in SR cancer compared to other colorectal cancer subtypes. COX-2 overexpression was found to be more likely to demonstrate a poor response to chemoradiotherapy^40^. Overall, the high expression of p53, EGFR, and COX-2 in AC results in poor response to chemoradiotherapy, and may confer early and late toxicity from RT, affecting patients’ long-term health-related quality of life. However, the previous statements were speculative, and further experimental studies are needed to better understand the mechanisms behind the variation in the effects of RT across different histological subtypes of CRC.

The current study's strength is that it offers a unique perspective on the possible link between RT and the prognosis of various CRC histological subtypes. Other strengths included the enormous amount of data on CRC patients, which allows for accurate extrapolation of the findings. In addition, competing risk regressions were used to confirm our findings, which improved the study's accuracy. On the other hand, the limitations must be acknowledged. First, SEER, in particular, is lacking in precise data on chemotherapy and RT. It does not distinguish between “unknown” and “no. ” Second, there was no information on the patient’s comorbidities, family history, body measures, clinical exams, or laboratory testing, which might have been associated with CRC outcomes and survival. Third, targeted therapy and immunotherapy are becoming more common in clinical practice for the treatment of CRC, However, due to the limited data, we could not assess the effect of targeted therapy and immunotherapy on the association of RT with the risk of CRC-related death. Forth, pathologic data was collected from a variety of hospitals and not submitted to a centralized review. Fifth, this is an observational study, therefore, while associations and correlations can be identified, it is not possible to establish a cause-and-effect relationship between variables solely based on observational data. Sixth, RT significantly reduced the risk of death in SR patients but not in MC patients. Nevertheless, a biological explanation for this phenomenon could not be identified in our study. Further investigations with longer follow-up and broader assessment of covariates are necessary to enhance our understanding of CRC subtypes and their responses to RT.

## Conclusions

The results of this retrospective cohort analysis revealed that the effect of RT varied according to CRC histological subtypes. This can ultimately lead to more personalized and effective treatment strategies for CRC patients. Future research on CRC is required in several areas, including molecular pathology, risk factors, genetic contributions, and diagnostic and therapeutic techniques.

## Supplementary Information

Supplementary Information.

## Data Availability

The data were abstracted from the Surveillance, Epidemiology, and End Results (SEER) database. This is an open database. (https://seer.cancer.gov).
