# Supplementary material for: The prognostic ability of radiotherapy of different colorectal cancer histological subtypes and tumor sites
Source: Sci Rep. 2023 Jul 20;13:11758. doi: 10.1038/s41598-023-38853-9 (PMC10359278; doi:10.1038/s41598-023-38853-9)

**Supplementary Materials**

**Supplementary Table 1. Baseline characteristics of the population stratified with and without radiotherapy for each pathological type.**

|  | **Adenocarcinoma** | | ***P*-value** | **Mucinous** | | ***P*-value** | **Signet-Ring Cell** | | ***P*-value** |
| --- | --- | --- | --- | --- | --- | --- | --- | --- | --- |
|  | **No-radiotherapy** | **Radiotherapy** |  | **No-radiotherapy** | **Radiotherapy** |  | **No-radiotherapy** | **Radiotherapy** |  |
| **n** | 45089 | 9085 |  | 3408 | 405 |  | 574 | 90 |  |
| **Age (Year)** | 65.23(13.42) | 60.10(12.82) | <0.001 | 65.31(14.34) | 58.90(14.23) | <0.001 | 63.28(15.44) | 54.62(15.86) | <0.001 |
| **Race (%)** |  |  | <0.001 |  |  | 0.119 |  |  | 0.067 |
| **White** | 33392(74.1) | 7047(77.6) |  | 2693(79.0) | 32(79.8) |  | 462(80.5) | 63(70.0) |  |
| **Black** | 5650(12.5) | 784(8.6) |  | 365(10.7) | 32(7.9) |  | 53(9.2) | 14(15.6) |  |
| **Others** | 6047(13.4) | 1254(13.8) |  | 350(10.3) | 50(12.3) |  | 59(10.3) | 13(14.4) |  |
| **Marital status (%)** |  |  | <0.001 |  |  | 0.002 |  |  | 0.001 |
| **Single** | 7356(16.3) | 1736(19.1) |  | 549(16.1) | 84(20.7) |  | 115(20.0) | 32(35.6) |  |
| **Married** | 23486(52.1) | 4956(54.6) |  | 1794(52.6) | 226(55.8) |  | 289(50.3) | 43(47.8) |  |
| **Other** | 14247(31.6) | 2393(26.3) |  | 1065(31.2) | 95(23.5) |  | 170(29.6) | 15(16.7) |  |
| **Sex (%)** |  |  | <0.001 |  |  | <0.001 |  |  | <0.001 |
| **Female** | 22032(48.9) | 3489(38.4) |  | 1793(52.6) | 162(40.0) |  | 300(52.3) | 26(28.9) |  |
| **Male** | 23057(51.1) | 5596(61.6) |  | 1615(47.4) | 243(60.0) |  | 274(47.7) | 64(71.1) |  |
| **Location (%)** |  |  | <0.001 |  |  | <0.001 |  |  | <0.001 |
| **Right** | 20570(45.6) | 261(2.9) |  | 2395(70.3) | 35(8.6) |  | 404(70.4) | 6(6.7) |  |
| **Left** | 14632(32.5) | 415(4.6) |  | 634(18.6) | 28(6.9) |  | 102(17.8) | 4(4.4) |  |
| **Rectum** | 8605(19.1) | 8356(92.0) |  | 252(7.4) | 340(84.0) |  | 43(7.5) | 80(88.9) |  |
| **Unknown** | 1282(2.8) | 53(0.6) |  | 127(3.7) | 2(0.5) |  | 25(4.4) | 0(0.0) |  |
| **Grade (%)** |  |  | <0.001 |  |  | <0.001 |  |  | 0.019 |
| **Well-differentiated** | 3397(7.5) | 494(5.4) |  | 434(12.7) | 23(5.7) |  | 3(0.5) | 3(3.3) |  |
| **Moderately-differentiated** | 29798(66.1) | 6018(66.2) |  | 1907(56.0) | 215(53.1) |  | 28(4.9) | 3(3.3) |  |
| **Poorly-differentiated** | 5930(13.2) | 872(9.6) |  | 497(14.6) | 79(19.5) |  | 362(63.1) | 52(57.8) |  |
| **Undifferentiated** | 1077(2.4) | 116(1.3) |  | 164(4.8) | 15(3.7) |  | 89(15.5) | 10(11.1) |  |
| **Unknown** | 4887(10.8) | 1585(17.4) |  | 406(11.9) | 73(18.0) |  | 92(16.0) | 22(24.4) |  |
| **AJCC 7th, T Stage (%)** |  |  | <0.001 |  |  | <0.001 |  |  | 0.319 |
| **T1** | 7217(16.0) | 473(5.2) |  | 118(3.5) | 12(3.0) |  | 23(4.0) | 4(4.4) |  |
| **T2** | 4106(9.1) | 643(7.1) |  | 243(7.1) | 11(2.7) |  | 18(3.1) | 4(4.4) |  |
| **T3** | 14024(31.1) | 4224(46.5) |  | 1215(35.7) | 187(46.2) |  | 131(22.8) | 27(30.0) |  |
| **T4** | 4953(11.0) | 1004(11.1) |  | 857(25.1) | 92(22.7) |  | 222(38.7) | 25(27.8) |  |
| **Unknown** | 14789(32.8) | 2741(30.2) |  | 975(28.6) | 103(25.4) |  | 180(31.4) | 30(33.3) |  |
| **AJCC 7th, N Stage (%)** |  |  | <0.001 |  |  | <0.001 |  |  | 0.672 |
| **N0** | 19127(42.4) | 2779(30.6) |  | 1403(41.2) | 104(25.7) |  | 135(23.5) | 17(18.9) |  |
| **N1** | 8013(17.8) | 2912(32.1) |  | 625(18.3) | 118(29.1) |  | 96(16.7) | 17(18.9) |  |
| **N2** | 4398(9.8) | 842(9.3) |  | 474(13.9) | 85(21.0) |  | 183(31.9) | 27(30.0) |  |
| **Unknown** | 13551(30.1) | 2552(28.1) |  | 906(26.6) | 98(24.2) |  | 160(27.9) | 29(32.2) |  |
| **AJCC 7th, M Stage (%)** |  |  | <0.001 |  |  | <0.001 |  |  | 0.065 |
| **M0** | 25944(57.5) | 5561(61.2) |  | 1869(54.8) | 261(64.4) |  | 252(43.9) | 47(52.2) |  |
| **M1** | 7278(16.1) | 1152(12.7) |  | 738(21.7) | 55(13.6) |  | 192(33.4) | 19(21.1) |  |
| **Unknown** | 11867(26.3) | 2372(26.1) |  | 801(23.5) | 89(22.0) |  | 130(22.6) | 24(26.7) |  |
| **Stages** |  |  | <0.001 |  |  | <0.001 |  |  | 0.152 |
| **I** | 7610(16.9) | 1827(20.1) |  | 276(8.1) | 11(2.7) |  | 20(3.5) | 4(4.4) |  |
| **II** | 8242(18.3) | 3066(33.7) |  | 834(24.5) | 76(18.8) |  | 60(10.5) | 8(8.9) |  |
| **III** | 7278(16.1) | 1152(12.7) |  | 728(21.4) | 167(41.2) |  | 160(27.9) | 33(36.7) |  |
| **IV** | 12955(28.7) | 2516(27.7) |  | 738(21.7) | 55(13.6) |  | 192(33.4) | 19(21.1) |  |
| **Unknown** | 7610(16.9) | 1827(20.1) |  | 832(24.4) | 96(23.7) |  | 142(24.7) | 26(28.9) |  |
| **CEA (%)** |  |  | <0.001 |  |  | <0.001 |  |  | 0.092 |
| **Negative** | 14200(31.5) | 3266(35.9) |  | 916(26.9) | 137(33.8) |  | 151(26.3) | 26(28.9) |  |
| **Positive** | 12327(27.3) | 3406(37.5) |  | 1126(33.0) | 143(35.3) |  | 210(36.6) | 41(45.6) |  |
| **Unknown** | 18562(41.2) | 2413(26.6) |  | 1366(40.1) | 125(30.9) |  | 213(37.1) | 23(25.6) |  |
| **Perineural Invasion (%)** |  |  | <0.001 |  |  | <0.001 |  |  | 0.179 |
| **Negative** | 32535(72.2) | 6105(67.2) |  | 2234(65.6) | 289(71.4) |  | 240(41.8) | 47(52.2) |  |
| **Positive** | 4041(9.0) | 857(9.4) |  | 240(7.0) | 53(13.1) |  | 110(19.2) | 14(15.6) |  |
| **Unknown** | 8513(18.9) | 2123(23.4) |  | 934(27.4) | 63(15.6) |  | 224(39.0) | 29(32.2) |  |
| **Liver Metastases (%)** |  |  | <0.001 |  |  | <0.001 |  |  | 0.057 |
| **No** | 36579(81.1) | 7978(87.8) |  | 2961(86.9) | 381(94.1) |  | 521(90.8) | 79(87.8) |  |
| **Yes** | 7500(16.6) | 1041(11.5) |  | 383(11.2) | 24(5.9) |  | 38(6.6) | 11(12.2) |  |
| **Unknown** | 1010(2.2) | 66(0.7) |  | 64(1.9) | 0(0.0) |  | 15(2.6) | 0(0.0) |  |
| **Bone Metastases (%)** |  |  | 0.003 |  |  | <0.001 |  |  | 0.049 |
| **No** | 43569(96.6) | 8771(96.5) |  | 3320(97.4) | 390(96.3) |  | 552(96.2) | 85(94.4) |  |
| **Yes** | 372(0.8) | 244(2.7) |  | 21(0.6) | 15(3.7) |  | 11(1.9) | 5(5.6) |  |
| **Unknown** | 1148(2.5) | 70(0.8) |  | 67(2.0) | 0(0.0) |  | 11(1.9) | 0(0.0) |  |
| **Brain Metastases (%)** |  |  | 0.020 |  |  | 0.002 |  |  | 0.013 |
| **No** | 43881(97.3) | 8891(97.9) |  | 3339(98.0) | 403(99.5) |  | 564(98.3) | 88(97.8) |  |
| **Yes** | 54(0.1) | 118(1.3) |  | 3(0.1) | 2(0.5) |  | 1(0.2) | 2(2.2) |  |
| **Unknown** | 1154(2.6) | 76(0.8) |  | 66(1.9) | 0(0.0) |  | 9(1.6) | 0(0.0) |  |
| **Lung Metastases (%)** |  |  | <0.001 |  |  | 0.003 |  |  | 0.042 |
| **No** | 41456(91.9) | 8471(93.2) |  | 3220(94.5) | 385(95.1) |  | 550(95.8) | 85(94.4) |  |
| **Yes** | 2431(5.4) | 528(5.8) |  | 114(3.3) | 20(4.9) |  | 11(1.9) | 5(5.6) |  |
| **Unknown** | 1202(2.7) | 86(0.9) |  | 74(2.2) | 0(0.0) |  | 13(2.3) | 0(0.0) |  |
| **Surgery (%)** |  |  | <0.001 |  |  | 0.498 |  |  | 0.024 |
| **No** | 6761(15.0) | 2022(22.3) |  | 326(9.6) | 44(10.9) |  | 118(20.6) | 30(33.3) |  |
| **Yes** | 38188(84.7) | 7060(77.7) |  | 3076(90.3) | 361(89.1) |  | 455(79.3) | 60(66.7) |  |
| **Unknown** | 140(0.3) | 3 (0.0) |  | 6(0.2) | 0(0.0) |  | 1(0.2) | 0(0.0) |  |
| [**Chemotherapy**](javascript:;) |  |  | <0.001 |  |  | <0.001 |  |  | <0.001 |
| **No/ Unknown** | 28495(63.2) | 600(6.6) |  | 1848(54.2) | 21(5.2) |  | 233(40.6) | 7(7.8) |  |
| **Yes** | 16594(36.8) | 8485(93.4) |  | 1560(45.8) | 384(94.8) |  | 341(59.4) | 83(92.2) |  |

**Note: The definitions of T, N, M were referred to as pathologic stage groups (pTNM). CEA: carcinoembryonic antigen.**

**Supplementary Figure 1. The distribution of propensity score of treated and control groups in PSM analysis.**


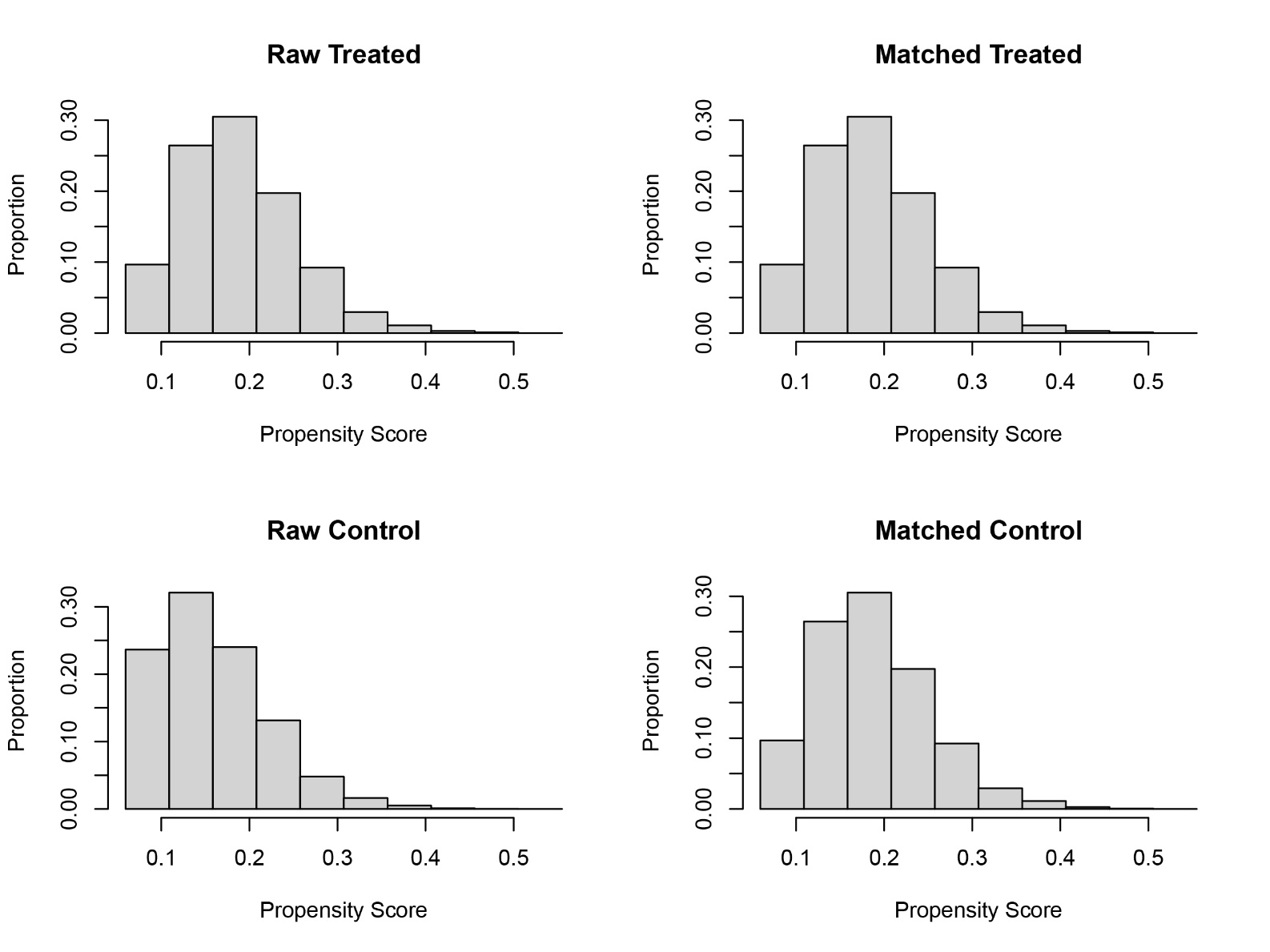

Supplement: Supplementary file 1 — Supplementary Information. [file 41598_2023_38853_MOESM1_ESM.docx]
